# Characteristics, potential sources, and cancer risk apportionment of PM_10_-bound polycyclic aromatic hydrocarbons in Bengbu, Central China

**DOI:** 10.3389/fpubh.2024.1445782

**Published:** 2024-11-08

**Authors:** Danchen Wu, Zhijing Ma, Haitao Diao, Wanru Wang, Liu Chen, Dalin Zhou, Jing Yang, Quan Zhen

**Affiliations:** School of Public Health, Bengbu Medical University, Bengbu, China

**Keywords:** PM10, polycyclic aromatic hydrocarbon, source analysis, positive matrix factorization, HYSPLIT model

## Abstract

Polycyclic aromatic hydrocarbons (PAHs) were measured in 342 daily PM_10_ samples collected in four seasons at a site in Bengbu, China. This study was a qualitative and quantitative investigation of the emission sources of atmospheric PAHs in Bengbu and the spatial distribution of regional PAH sources in PM_10_ samples. The annual concentrations of the 16 EPA priority PAHs ranged from 1.45 to 62.16 ng/m^3^, with an annual mean of 7.63 ± 7.38 ng/m^3^. The seasonal trends during the year were: winter (6.13–62.16 ng/m^3^, median = 14.99 ng/m^3^) > autumn (2.01–18.78 ng/m^3^, median = 4.90 ng/m^3^) > spring (1.45–19.34 ng/m^3^, median = 3.32 ng/m^3^) > summer (1.57–4.27 ng/m^3^, median = 2.12 ng/m^3^). The PAHs over the year were dominated by medium-molecular-weight PAHs (39.81%), followed by high-molecular-weight PAHs (35.77%), and low-molecular-weight PAHs (24.42%). The diagnostic ratio method and positive matrix factorization revealed that the PAH sources in Bengbu in spring and summer were industrial emissions, coal and biomass combustion, and traffic emissions; while the sources in autumn and winter were coal and biomass combustion and traffic emissions. According to a backward trajectory clustering analysis and potential source contribution function analysis, Bengbu City was mainly affected by pollution from the northern and northwestern regions in spring, autumn, and winter, while it was more affected by the coastal monsoon in summer. The PAH pollution in Bengbu was most severe in spring, autumn, and winter, and the health risk to the population was also most severe at that time. The health risk to adult males (3.35 × 10^−4^) was greater than the risk to adult females (3.14 × 10^−4^), and the health risk to adults was greater than the risk to children (2.52 × 10^−4^).

## 1 Introduction

Polycyclic aromatic hydrocarbons (PAHs) are hydrocarbons consisting of two or more benzene rings linked together in a fused ring. They are widely distributed in various media, such as the atmosphere, water, and soil (1). Although PAHs occur only in trace amounts in the environment, they have been identified by the United States Environmental Protection Agency (USEPA) and the European Union (EU) as priority pollutants for environmental control because of their toxicity and potential for bioconcentration, and they present a serious risk to ecosystems and human health (2). Studies have shown that PAHs can attach to airborne particles and accumulate in the body through inhalation, ingestion, or absorption through skin contact, leading to poisoning, anemia, neurotoxicity, lung cell damage, and other respiratory problems (3). Polycyclic aromatic hydrocarbons can also cause serious health problems such as birth defects, genetic damage, and lung and skin cancer (4).

Several researchers have already conducted studies on PAHs in atmospheric particulate matter and have identified seasonal trends. In a study of atmospheric PAHs in Shanghai, Chen Y et al. (5) derived annual PAH concentrations of 167 ± 109 ng/m^3^ in an urban area and 216 ± 86.5 ng/m^3^ in a suburban area. The same seasonal trend was observed at both sites, with the highest concentrations occurring in winter and the lowest in autumn. In a study in the coastal area of Dalian (6), the average PAH concentrations in air and seawater were 27.5 ± 14.6 ng/m^3^ and 49.5 ± 20.5 ng/L, respectively. The PAH concentrations in air were seasonal, being higher in winter (99 ng/m^3^) than in summer (2.7 ng/m^3^). Many studies of airborne PAHs, including those referred to above, have focused on large cities, while the effects of atmospheric PAH pollution in small and medium-sized towns have not received as much attention.

Polycyclic aromatic hydrocarbons from the incomplete combustion of organic matter (such as coal, oil, and natural gas) and the pyrolysis of large organic compounds dominate atmospheric emissions (7). To better understand and control PAH pollution processes, several methods have been developed to identify PAH sources. Diagnostic PAH ratios (8) and positive matrix factorization (PMF) (9) are common methods applied for pollution source analysis. Ali-Taleshi MS et al. (10) used these two methods in their study in the Tehran region of Iran and found that industrial and petroleum emissions contributed the most to overall PAH pollution during the non-heating period (19.8 and 27.2%, respectively), while vehicle exhaust and biomass-fired natural gas emissions contributed the most during the heating period (40.7 and 29.6%, respectively). The application of diagnostic PAH ratios and PMF modeling by Chao S et al. (11) showed that the largest contribution to PAH emissions in downtown Beijing came from vehicle emissions (54.6%), followed by coal combustion (29.8%). The combination of the two source analysis techniques makes the source results more reliable by validating each other, although this has not been commonly undertaken in small and medium-sized city studies.

In addition to the sources of emissions, the long-range transport of air pollutants is also a matter of great concern. Duan L et al. (12) found that the Yangtze River Delta region was the main PAH source area in Hangzhou in spring and summer, while in autumn and winter PAH pollution mainly originated from long-range transport from northern China. Zhang Y et al. (13) showed that the contribution of long-distance transport from northern Wuhan to the north and east of China was greater than that from southern China, while the contributions from local areas were higher than that from long-range transport. In the analysis of aerosol samples from the Chongqing metropolitan area (14), a potential source contribution function (PSCF) analysis was used to identify south-eastern Sichuan and north-western Chongqing as the main potential PAH sources. Therefore, the identification of source areas using a cluster analysis and the PSCF method was necessary to improve the management of pollutant emissions in each region and to achieve the joint regional management of the atmosphere across regions. Many studies have not addressed the long-range transport of atmospheric pollution when investigating PAH sources.

Bengbu city is located in the middle reaches of the Huai River, with a total area of 969.39 km^2^ and a total population over 800,000 within its urban area. This makes it one of China’s small and medium-sized cities. (15). It is situated in the middle of Anhui Province and is an important node of the East China Economic Zone and the Yangtze River Delta Economic Circle. This geographical location makes Bengbu an important transport hub connecting East and Central China, and Bengbu has a relatively well-developed industrial base and economic system compared to other locations in eastern China. It is therefore considered a typical small to medium-sized city in the Yangtze River Delta region (16). In recent years, the Yangtze River Delta region has experienced frequent and severe haze (17). Atmospheric studies in the Yangtze River Delta region have focused on large cities such as Shanghai (18), Nanjing (19), and Hefei (20), and have not yet investigated the small and medium-sized cities. Based on recent environmental monitoring data, Bengbu ranks poorly in air quality compared to other areas within Anhui Province (21). Quantitative source identification related to atmospheric PAHs was considered in this study for a more precise understanding of pollution emissions. To determine the spatial distribution of pollutant sources, we used the backward trajectory method of airflow to trace the atmospheric transport of pollutants. We also conducted a potential source analysis to identify the areas with the greatest impact on pollution in Bengbu City.

The main objectives of this study were to: (1) analyze the changes in atmospheric PAHs concentrations as well as the season and molecular structure (i.e., number of rings) trends in Bengbu over a whole year; (2) investigate the specific PAH sources in Bengbu using a combination of the qualitative diagnostic PAH ratios method and a quantitative PMF model; (3) trace all the pollutant streams entering Bengbu City using the HYSPLIT model to identify the areas in Bengbu City with high levels of pollution through a potential source analysis; (4) assess the health risks of PAHs to the population of Bengbu City, we also calculated the possible health risks of air pollution on adult males, adult females, and children. These data provide important information for policy makers to develop emission reduction strategies to reduce PAH emissions and improve air quality in Bengbu and other similar small and medium-sized cities in China.

## 2 Experimental materials and analysis

### 2.1 Sampling site and sampling

The sampling site was located on the rooftop of a teaching building at Bengbu Medical University (117.433°E, 32.908°N) at a height of about 15 m above ground level. The sampling duration was from October 2021 to September 2022. All PM_10_ samples were collected using a high-flow particulate sampler (Model 2031, Laoshan Institute of Applied Technology, Qingdao, China) with a flow rate of 1.05 m^3^/min and a daily sampling period of 23 h. Sampling times were fixed, from 9:00 a.m. each day to 8:00 a.m. the next day. A total of 342 samples were collected. Samples were collected on glass fiber membranes (20 × 25 cm, Shanghai Xingya Purification Material Factory), which were placed in a muffle furnace at 400°C for 4 h prior to sampling to remove organic matter. After sampling, the membranes were folded and sealed in aluminum foil envelopes and then stored in a refrigerator at −20°C prior to analysis. The meteorological parameters observed during the study period are shown in Table 1.

**Table 1:** Variation in meteorological parameters observed during the study period.

Season	Temperature (°C)			Relative humidity (%)	Wind speed (mph)	Precipitation (mm)
	Min	Max	Average			
Spring	5.63	23.71	16.45	63.69 ± 20.24	2.84 ± 0.42	0.08 ± 0.04
Summer	19.25	37.81	27.89	74.19 ± 11.74	2.52 ± 0.48	1.25 ± 0.04
Autumn	5.11	17.61	15.94	73.32 ± 15.11	1.62 ± 0.31	0.24 ± 0.03
Winter	−1.41	10.12	3.41	67.78 ± 9.76	2.03 ± 0.60	0.54 ± 0.09

### 2.2 Analysis of PAHs

The method described in the “Determination of polycyclic aromatic hydrocarbons in ambient air and exhaust gas phase and particulate matter by gas chromatography–mass spectrometry (HJ 646–2013)” was used for the analysis of PAHs, with a slight modification (22). Each PM_10_ sample was placed in a 20 mL sample bottle and a dichloromethane (≥99.9%, Shanghai Aladdin Biochemical Technology Co., China)/hexane (95%, Shanghai Aladdin Biochemical Technology Co., China; 1:1, V/V) solution was added until the filter membrane was completely immersed. The sample bottle was ultrasonically extracted twice for 10 min using an ultrasonic cleaner. After ultrasound, the supernatant was extracted into a sharp-bottomed tube. Then, the sharp-bottomed tube was placed on a water bath nitrogen blowing apparatus to concentrate the solution. Then n-hexane and the internal standard (naphthalene-d8, acenaphthene-d10, phenanthrene-d10, chrysene-d12, and perylene-d12, Shanghai Anpu Experimental Technology Co.) were added and the solution was transferred to a gas chromatography vial (23).

Samples were analyzed by gas chromatography–mass spectrometry (5975C-7890A, Agilent, Santa Clara, CA, United States). A column (30 m × 0.25 mm × 0.25 μm; DB-5MS, Agilent) was used to quantify PAHs and 1 μL of the sample was injected in splitless mode. The MS was operated in electron impact mode at 70 eV. The selected ion monitoring mode was used to collect the data. Helium was used as the carrier gas at a flow rate of 0.5 mL/min (24). The temperature program of the GC oven was 100°C and held for 2 min at the first stage, then increased to 320°C at rate of 10°C/min and kept for 5 min. The 16 USEPA priority PAHs are: naphthalene (Nap), acenaphthylene (Acy), acenaphthene (Ace), fluorene (Flu), phenanthrene (Phe), anthracene (An), fluoranthene (Fl), pyrene (Pyr), benzo(a)anthracene (BaA), chrysene (Chr), benzo(b)fluoanthene (BbF), benzo(k)fluoranthene (BkF), benzo(a)pyrene (BaP), indeno (1,2,3-cd) pyrene (IcP), dibenzo (a, h)anthracene (DBA), benzo (g, h, i)perylene (BghiP) (25).

### 2.3 Quality assurance and quality control

The atmospheric samplers underwent a routine flow rate calibration to ensure precise sampling, and strict quality assurance and control measures were adopted for all analytical procedures. Throughout the experimental procedures, meticulous quality control and assurance were maintained with the use of method blanks, program blanks, and standard spiked recovery samples during the testing phase. In the detection process, the correlation coefficient for the standard curve of the 16 priority PAHs exceeded 0.99. A blank filter was placed the power-off sampler and treated the same as a normal sampling filter. The relative deviation of PAHs in parallel samples was rigorously controlled to be below 15%, accompanied by a sample repeatability of 10%. A blank was added after every three samples in the GC–MS procedure to check for sample contamination. Deuterated PAHs (naphthalene-d8, acenaphthene-d10, phenanthrene-d10, chrysene-d12, and perylene-d10, US o2si smart solution) were added to the samples as recovery substitutes for detection, and the recoveries were within the acceptable range of 74.38 to 128.42%. The detection limits (ng/m^3^) were: 0.026 (Nap), 0.008 (Acy), 0.008 (Ace), 0.011 (Flu), 0.015 (Phe), 0.031 (An), 0.014 (Fl), 0.033 (Pyr), 0.029 (BaA), 0.030 (Chr), 0.021 (BbF), 0.013 (BkF), 0.015 (BaP), 0.015 (IcP), 0.003 (DBA), and 0.009 (BghiP).

### 2.4 Source apportionment of PAHs

#### 2.4.1 The PAH diagnostic ratios

Diagnostic ratios are widely used to identify and assess PAH emission sources (26) The Flu/(Flu + Pyr), Ant/(Ant + Phe), BaA/(BaA + Chr), and InP/(InP + BghiP) diagnostic ratios were used in this study (27).

#### 2.4.2 Positive matrix factorization modeling

The USEPA PMF 5.0 model was used for the qualitative and quantitative identification of PAH sources. It is a receptor-based source apportionment model, which is recommended by the USEPA (28). The uncertainty was calculated from the method detection limit (MDL) and determination error fraction. If the concentration was lower than MDL, the uncertainty was set to 5/6 of the MDL; if the concentration was higher than MDL, the uncertainty was set to the square root of the sum of squared error fraction-weighted concentration and squared MDL (29).

#### 2.4.3 The HYSPLIT model

We calculated backward trajectories using the HYSPLIT model.^1^ The model was run at starting times of 00:00, 06:00, 12:00, and 18:00 UTC every day during the sampling period. The duration of the calculation was 48 h and the height was 500 m above ground level. Daily meteorological data obtained from the Global Data Assimilation System was provided by the National Centers for Environmental Prediction. The HYPSPLIT model results were saved as endpoint files and then imported into the TrajStat software^2^ for trajectory clustering and statistics. The PSCF was determined using TrajStat to identify the spatial distribution of PAH sources.

### 2.5 Health risk assessment of PAHs

Inhalation is a crucial pathway for PAH exposure. The incremental life-time cancer risk (ILCR) model was used in this study to assess the carcinogenic risk of PAHs. This is an effective evaluation model based on BaP to assess human health risks. The calculation formula of the health risk assessment model is shown in Equation 1.


(1) $$\mathrm{ILCR}=\frac{CSF\times CA\times IR\times EF\times ED}{BW\times AT}$$

where, CSF is the inhalation cancer slope factor of BaP, 3.14 kg d mg^−1^; CA is the BaP equivalent (BaPeq) concentration, in mg/m^3^, and is calculated from the product of the concentration and the toxicity equivalency factor (TEF) of each PAH congener. The TEFs of the 16 priority PAHs are as follows: NaP, Ace, Acy, Fl, Phe, Flu, and Pyr = 0.001; Ant, BghiP, and Chry = 0.01; BaA, BbF, BkF, and InP = 0.1; and BaP and DahA = 1 (30, 31). The other parameters of human inhalation exposure (32) were as presented in Table 2.

**Table 2:** Human inhalation exposure parameters.

Factor	Definition	Unit	Values		
			Adult males	Adult females	Children
IR	Inhalation rate	m^3^/d	17.7	14.5	10.8
EF	Exposure frequency	d/a	365	365	365
ED	Exposure duration	a	30	30	18
BW	Average body weight	kg	66.1	57.8	32.2
AT	Average exposure time	d	25,550	25,550	25,550

### 2.6 Data analysis

Statistical analyses were performed using SPSS (IBM SPSS software 22.0), including a one-way analysis of variance (ANOVA), with statistical significance at *p* < 0.05; and mean, range, and variance of PAH concentrations. Origin 2023 software was used to produce graphs of PAH concentration changes and seasonal variations.

## 3 Results and discussion

### 3.1 Seasonal variation of PAH concentrations

**Figure 1:**
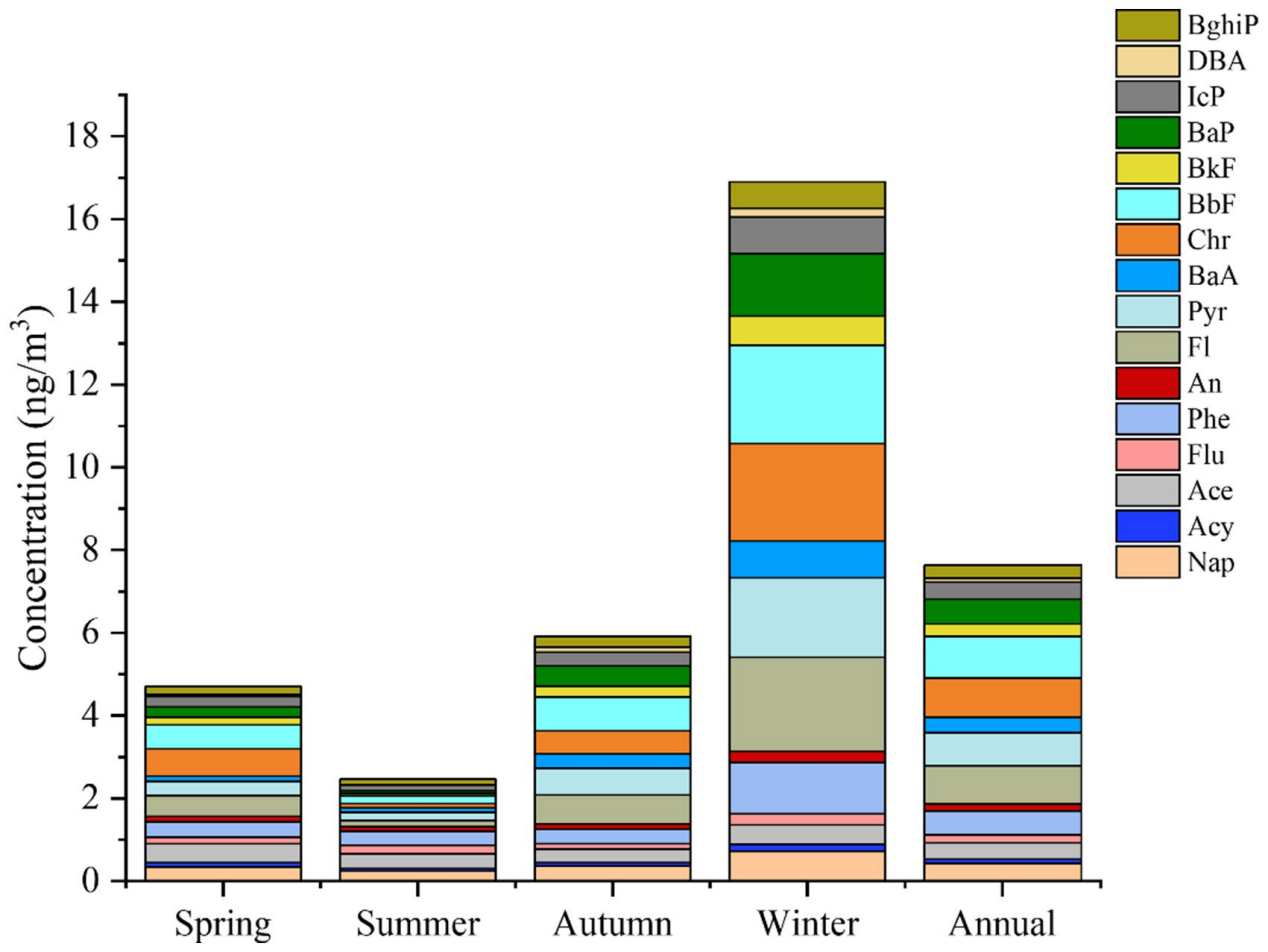
The concentrations of the 16 priority PHAs in different seasons.

The PAH concentrations over the whole year ranged from 1.45 to 62.16 ng /m^3^, the median was 4.36 ng/m^3^, with an annual mean concentration (± standard deviation, SD) of 7.63 ± 7.38 ng/m^3^. Among the 16 PAHs, Phe, Flt, Pyr, Chr, BbF, and BaP were the most abundant, accounting for 63.71% of the total PAHs, as shown in Figure 1. The overall PAH concentrations were higher than the observed PAH concentration in atmospheric PM_10_ in Sri Lanka (3.06–36.88 ng/m^3^) (33), Bangkok (35.80–55.50 ng/m^3^) (34), and Chengdu (12.25–58.56 ng/m^3^) (35); but lower than the PAH concentration in atmospheric PM_10_ in the Xinjiang region (11.98–138.46 ng/m^3^) (36), Hefei (17.70–78.26 ng/m^3^) (20) and Guangzhou (7.4–159.4 ng/m^3^) (37). This indicates an average level of PAH pollution in the Bengbu area.

In terms of seasonal trends, the PAH concentration was significantly higher in winter than in the other three seasons (one-way ANOVA, *p* < 0.01). No significant differences were observed among the other three seasons. Overall, the values decreased in the order of winter (6.13–62.16 ng/m^3^, median = 14.99 ng/m^3^) > autumn (2.01–18.78 ng/m^3^, median = 4.90 ng/m^3^) > spring (1.45–19.34 ng/m^3^, median = 3.32 ng/m^3^) > summer (1.57–4.27 ng/m^3^, median = 2.12 ng/m^3^). This trend was consistent with those reported in previous studies in the central Qilian Mountains (38) and Zaragoza, Spain (39).

Among the 16 PAH monomers, BaP presented a carcinogenic risk due to its frequency of occurrence, high toxicity, and potential threat to human health. In our study, its annual mean concentration was 0.61 ng/m^3^, accounting for 8.11% of ΣPAHs. The mean BaP concentration was much higher in winter (1.51 ng/m^3^) than in the other seasons (spring: 0.27 ng/m^3^; summer: 0.08 ng/m^3^; and autumn: 0.49 ng/m^3^). In this study, the mean winter *ρ*(BaP) concentration was 1.51 ng/m^3^ and did not exceed the secondary level (2.5 ng/m^3^) in China’s air quality standard (GB 3095–2012). The ρ(BaP) concentration in winter in Bengbu was 1/10 of the winter BaP concentration in Urumqi (10.94 ng/m^3^) (40), 1/14 of the winter concentration in Chengde (14.3 ng/m^3^) (41), and 1.11 times the winter concentration in Isfahan (1.35 ng/m^3^) (42), suggesting that the carcinogenic risk of BaP from respiratory exposure of the residents in the present study was lower than that in the Urumqi and Chengde regions and higher than that in the Isfahan region. During the winter sampling period there were 9 days when the BaP concentrations exceeded 2.5 ng/m^3^. Residents of Bengbu may therefore face a health risk from respiratory exposure to PAHs during the winter.

### 3.2 The PAH compositions

Polycyclic aromatic hydrocarbons can be separated into low-molecular-weight (LMW, 2–3 rings), middle-molecular-weight (MMW, 4 rings), and high-molecular-weight (HMW, 5–6 rings) congeners. The LMW and MMW PAHs are more volatile than the HMW PAHs and are generally present in both the particle and gaseous phases. The HMW PAHs are only present in the particle phase (43).

**Figure 2:**
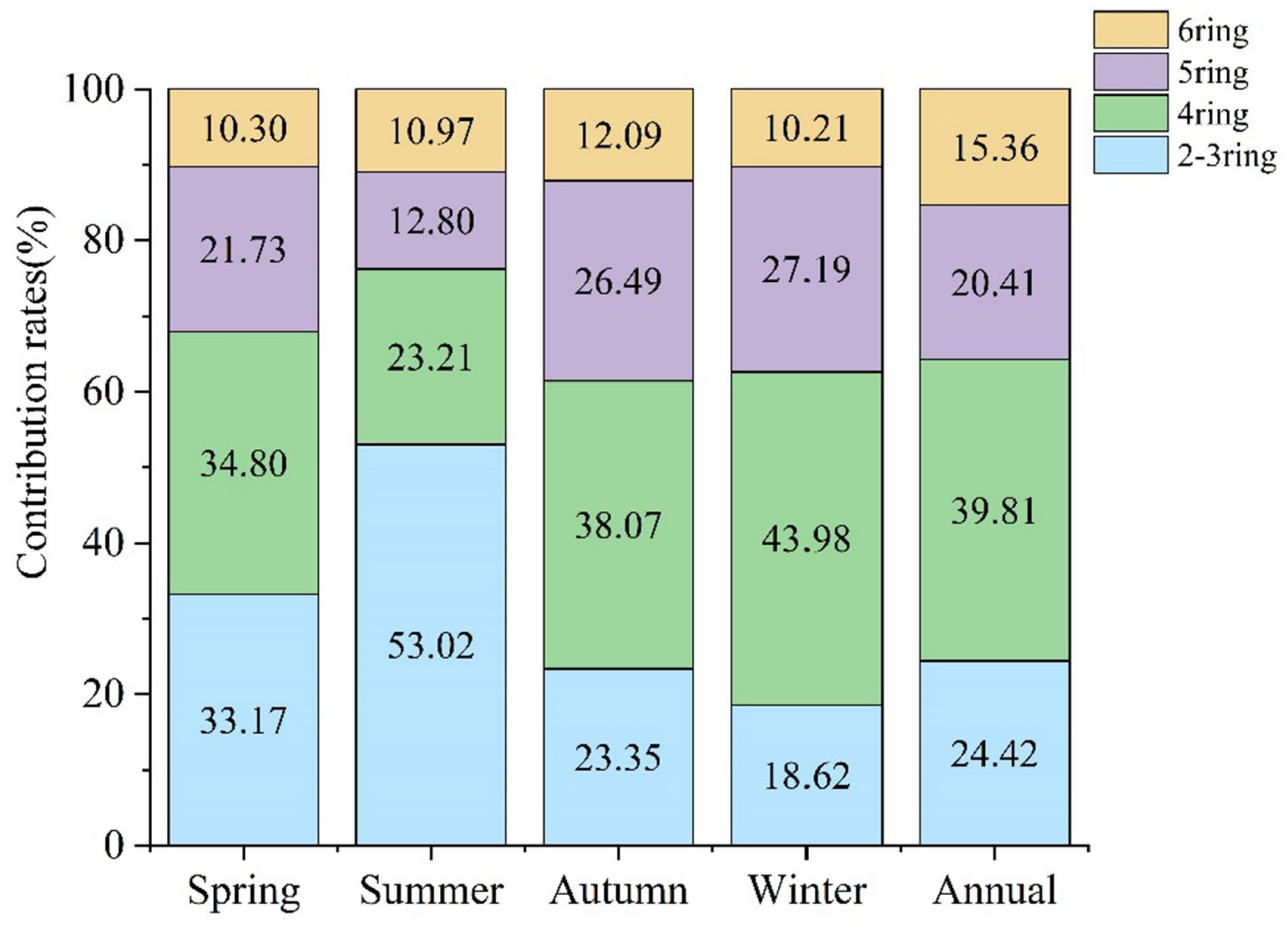
Contributions of PAHs with different ring numbers to the total PAH concentration in the four seasons.

The proportions of ΣPAHs in the four seasons were winter (56.50%) > autumn (19.78%) > spring (16.61%) > summer (7.11%). The PAHs over the full year were dominated by MMW PAHs (39.81%), followed by HMW PAHs (35.77%), and LMW PAHs (24.42%). The contribution of the PAHs of different molecular weights in the various seasons is shown in Figure 2. It can be seen that MMW and HMW PAHs accounted for the largest proportion (75.58%) of PAHs over the year. The highest proportions of MMW and HMW PAHs occurred in spring, autumn, and winter (66.81, 76.63, and 81.41%, respectively), while for LMW PAHs the highest proportion occurred during the summer months (53.01%). This could be due to PAHs tending to accumulate on particles during the colder seasons. In summer, the high temperature and relative humidity, together with high rainfall (Table 1) may have promoted the rapid degradation of PAHs, and also promoted strong photochemical reactions with atmospheric radicals and ozone (44).

### 3.3 Source apportionment of PAHs

#### 3.3.1 The PAH diagnostic ratios

The BaA/(BaA + Chr), Flu/(Flu+Pyr), Ant/(Ant+Phe), and InP/(InP + BghiP) ratios were calculated to investigate the PAH sources in Bengbu City, with the results shown in Table 3.

**Table 3:** Diagnostic ratios of PAHs in ambient PM_10_.

Diagnostic ratios	Value	Indicator source	References	Results			
				Spring	Summer	Autumn	Winter
Flu / (Flu +Pyr)	<0.4	Oil source	(57)	0.32 ± 0.16	0.49 ± 0.02	0.23 ± 0.14	0.13 ± 0.06
	0.4–0.5	Liquid fossil fuel combustion					
	>0.5	Coal and biomass combustion					
Ant / (Ant +Phe)	<0.1	Oil source	(26)	0.28 ± 0.05	0.27 ± 0.06	0.31 ± 0.06	0.19 ± 0.04
	>0.1	Combustion source					
BaA / (BaA + Chr)	<0.2	Oil source	(58)	0.13 ± 0.11	0.57 ± 0.16	0.45 ± 0.10	0.37 ± 0.05
	0.2–0.35	Oil or combustion sources					
	>0.35	Combustion source					
InP / (InP + BghiP)	<0.2	Oil source	(26)	0.57 ± 0.03	0.32 ± 0.02	0.56 ± 0.02	0.58 ± 0.02
	0.2–0.5	Liquid fossil fuel combustion					
	>0.5	Coal and biomass combustion					

The values of BaA/(BaA + Chr) in spring were in the range of 0.05–0.22, with mean values below 0.2, whereas the values of Flu/(Flu + Pyr) in autumn and winter were in the ranges of 0.09–0.39 and 0.05–0.23, respectively, with mean values below 0.4, indicating the contribution of petroleum sources in spring, autumn, and winter. Additionally, the values of InP/(InP + BghiP) were 0.51–0.63, 0.54–0.59, and 0.55–0.61 in spring, autumn, and winter, respectively. The average values were all >0.5, indicating that there was also a combination of contributions from coal and biomass combustion in spring, autumn, and winter. In contrast, the summer values of Flu/(Flu + Pyr) and InP/(InP + BghiP) were 0.46–0.51 and 0.28–0.35, respectively, with mean values of 0.49 and 0.32, indicating that the main PAH source in summer was the combustion of liquid fossil fuels. The average value of Ant/(Ant + Phe) over the four seasons was >0.1, demonstrating that combustion sources had an effect on PAH pollution in Bengbu City during the study period.

The results of the diagnostic ratios method showed that the PAH sources varied among the four seasons, with petroleum emissions, coal and biomass combustion, and liquid fossil fuel combustion being the main sources contributing to PAH pollution in Bengbu.

#### 3.3.2 The PMF model

**Figure 3:**
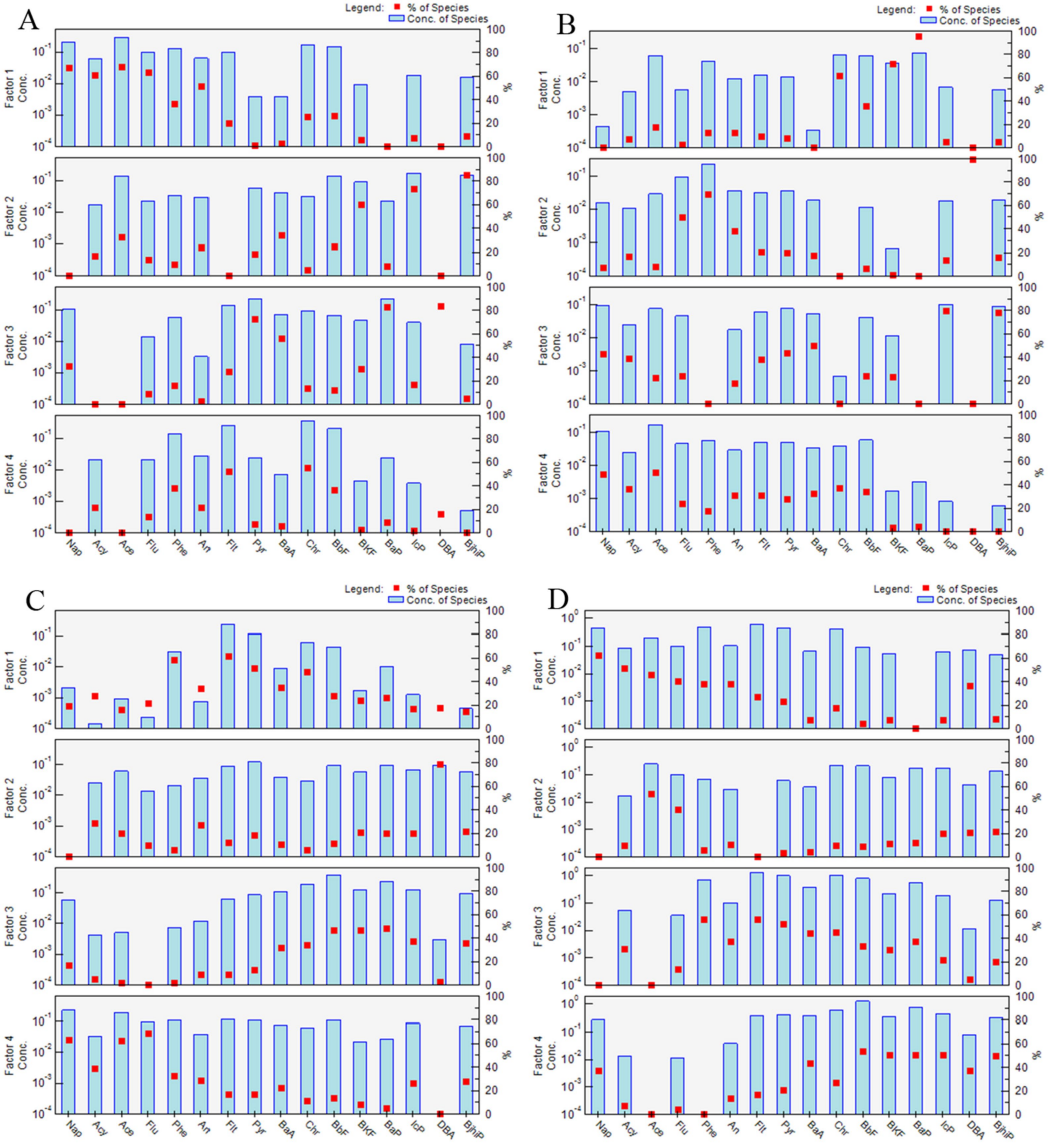
Source profiles and individual contributions of each factor to the PAH concentrations in the four seasons: (A) spring, (B) summer, (C) autumn, and (D) winter.

Four distinct PAH sources were identified in the four seasons (Figure 3).

Spring factor 1, summer and autumn factor 3, and winter factor 4 were highly loaded with Phe, Ant, Fl, Pyr, BaA, and Chr, which are major constituents of coal and biomass combustion emissions (45). Their contribution was larger in winter (42.39%) than in spring (21.46%), summer (20.34%), and autumn (24.94). The highest contributions of coal and biomass combustion occurred in the winter months, probably due to the high use of coal and biomass for heating (29) and occurrence of temperature inversions, which are not conducive to the diffusion of pollutants (46).

Benzo(a)pyrene is often considered to be a good marker of powered vehicles, while BkF is an important component of diesel-powered vehicles (47), and InP, DBA, BghiP, are typical markers of traffic emissions (48). Factor 3 in spring, factor 1 in summer, factor 4 in autumn, and factor 2 in winter were mainly composed of BaA, Chr, B(b/k) F, BaP, InP, DBA, and BghiP, which have been identified as markers of traffic emissions. Their contributions throughout the year were spring (12.29%), summer (35.78%), autumn (27.96%), and winter (18.40%). Their contribution was particularly prominent in summer, probably because the production of PAHs was enhanced under the high temperatures and intense light conditions of summer (41).

Factor 4 in spring, factor 2 in summer, factor 1 in autumn, and factor 3 in winter were predominantly weighted by BaP, followed by B(b/k) F, Chr, and Phe. These monomers are recognized as markers of the coking industry (29). Their contributions were spring (23.31%), summer (11.50%), autumn (21.17%), and winter (21.80%). The contributions of industrial emissions varied little among the four seasons, suggesting that industrial pollution emissions were relatively constant during the study period.

Low-molecular-weight PAHs are typical markers of the volatilization of crude oil and petroleum products (49). Factor 2 in spring, factor 4, in summer, factor 2 in autumn, and factor 1 in winter were mostly associated with Acy, Ace, Flu, and Phe. Therefore, the source may be emissions from the volatilization of crude oil and petroleum products. The contributions of the four seasons were spring (42.94%), summer (32.38%), autumn (25.93%), and winter (17.41%). The higher contributions in spring and summer than in autumn and winter were probably due to higher contributions from crude oil and oil volatiles as a result of the warmer temperatures (41).

From the PMF results it could be concluded that the PAH sources in spring and summer were industrial emissions, volatile emissions from crude oil and petroleum products, and traffic emissions, while the PAH sources in autumn and winter were coal and biomass combustion and industrial emissions. The PMF results were essentially the same as the diagnostic ratio method.

#### 3.3.3 The HYSPLIT model

**Figure 4:**
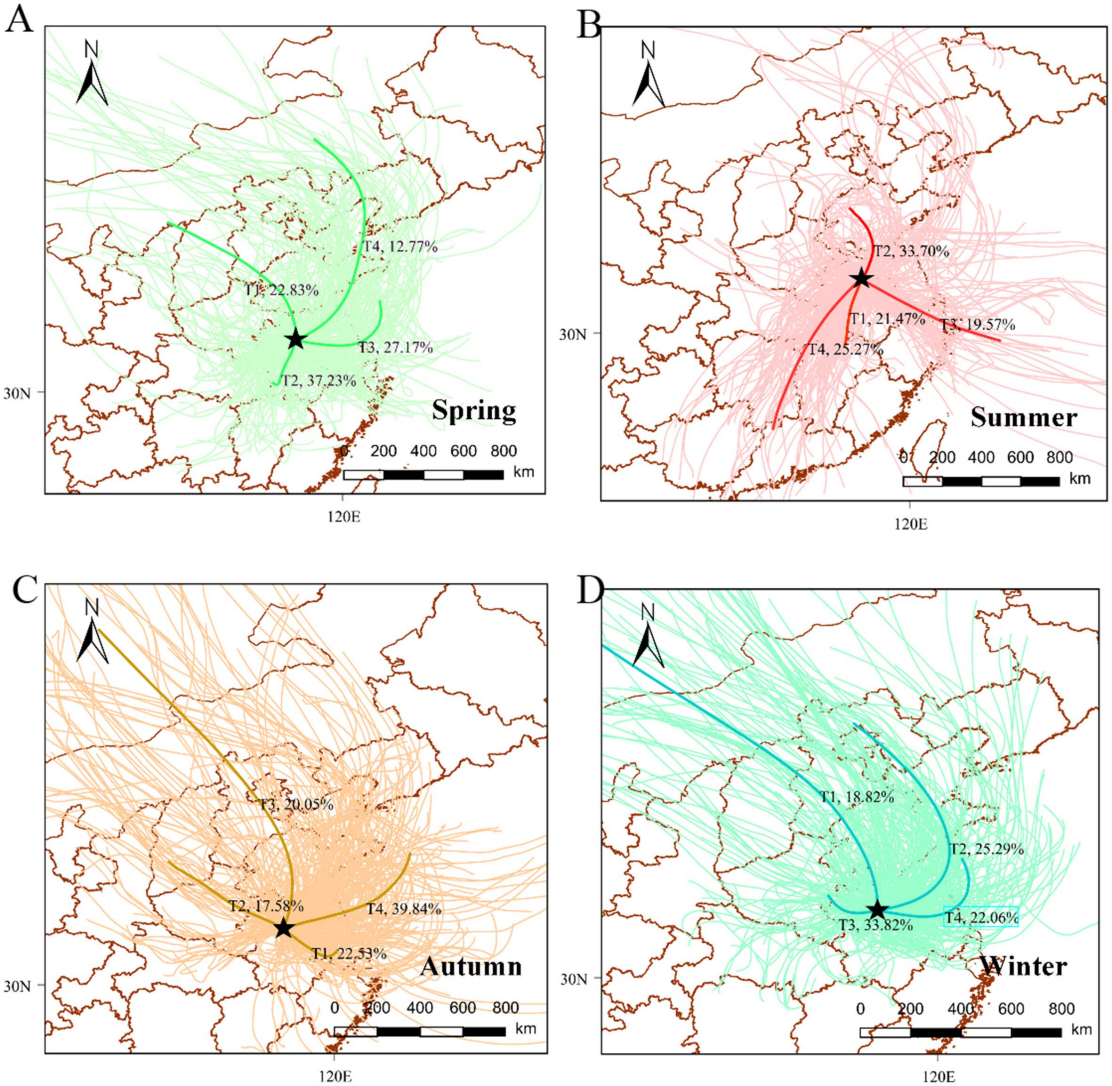
Cluster analysis of a 24 h backward trajectory of air masses during the study period: (A) spring, (B) summer, (C) autumn, and (D) winter.

**Figure 5:**
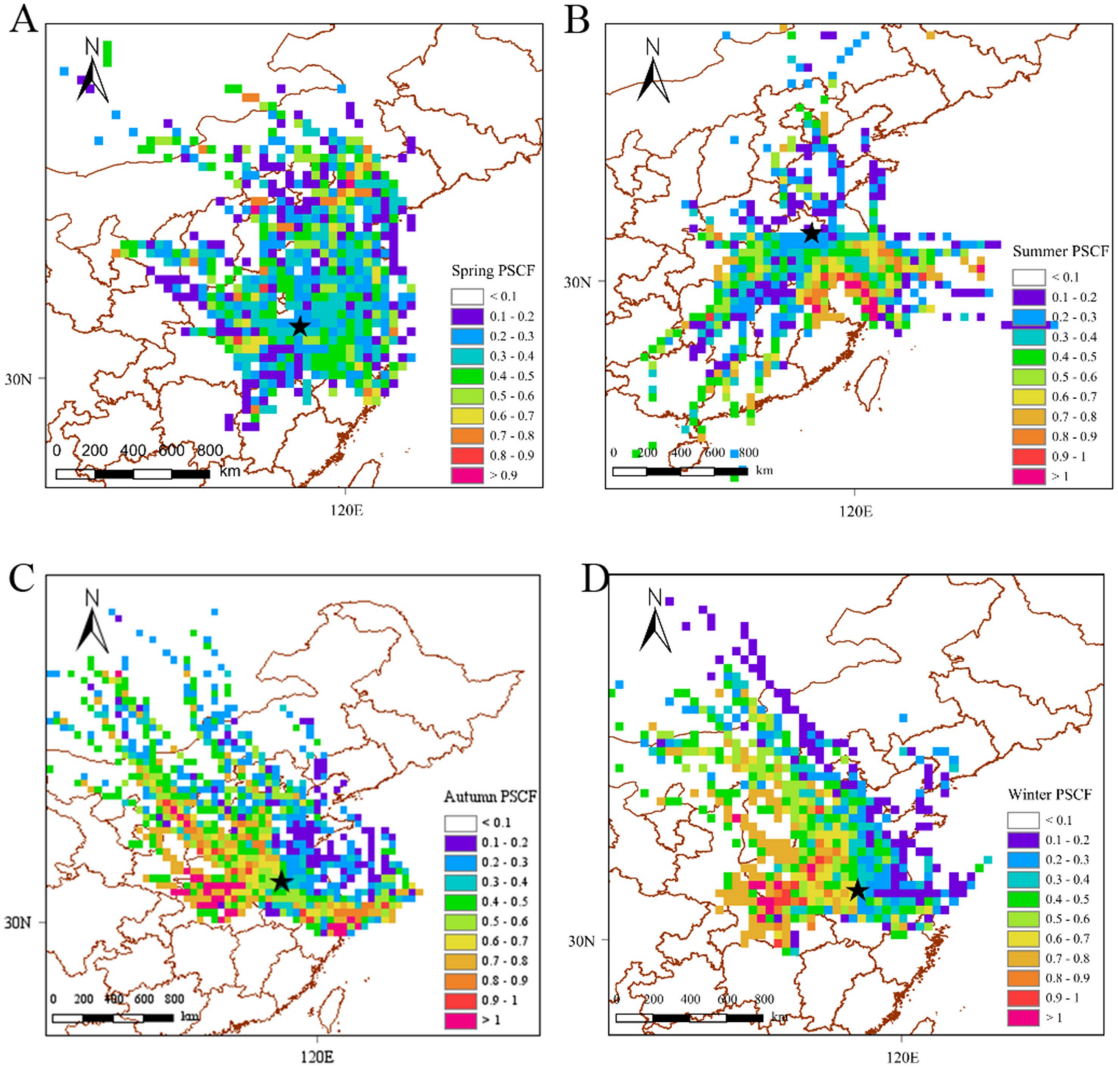
Potential PAH sources as determined by the PSCF algorithm: (A) spring, (B) summer, (C) autumn, and (D) winter.

Air mass trajectories during the four seasons were clustered based on the characteristics of the spatial distributions of the trajectories in each season. Figure 4 shows the clusters of the backward trajectories for each sampling season. Four distinct clusters were identified. The PSCF values and potential spatial distributions of PAH sources in Bengbu are presented in Figure 5. The color of the legend represents the potential pollution level: red represents high pollution levels, while blue represents low pollution levels. Pollution levels varied greatly from season to season due to the different trajectories of the air masses.

In spring, cloud T1 originates from the north-west (e.g., Shanxi and Henan) and cloud T4 originates from the north (e.g., Liaoning and Shandong). These clouds had high PAH concentrations of 5.31 and 5.01 ng/m^3^, respectively, which were higher than the spring PAH concentration in Bengbu (4.69 ng/m^3^). In contrast, the pollutant concentrations from the southern cloud T2 from Jiangxi and the eastern cloud T3 from Jiangsu (4.45 and 4.26 ng/m^3^, respectively) were lower than the average concentration in Bengbu in spring. The areas with high PSCF scores in the spring were in the north and north-west, including Shanxi and Shandong, which were identified as potentially polluted areas in the spring. Liaoning (46) and Shanxi (50), which are traditional industrial bases and coal provinces in China, have higher emissions in spring, and the northerly winds in spring facilitate the long-distance transport of pollutants from northern regions, which may exacerbate some of the industrial emissions in Bengbu City in spring.

Bengbu was most affected by pollution from air currents (cloud T3) from the east coast of Zhejiang and other areas during the summer months, with a PAH concentration of 2.79 ng/m^3^. This was followed by southern areas such as Fujian, where the pollution concentration was second only to the eastern areas (2.62 ng/m^3^). The pollutant concentrations carried by these two air trajectories exceeded the summer PAH concentration in Bengbu (2.46 ng/m^3^). The airflow from central Shandong (cloud T2) and the airflow from Hunan and Hubei (cloud T4) carried lower concentrations of pollutants than the summer concentrations in Bengbu, which were 2.28 ng/m^3^ and 2.27 ng/m^3^. The PSCF simulation also showed that the summer pollution zone was mainly located in the southeastern coastal area. Coastal regions such as Jiangsu (51), Zhejiang (12) and Shanghai (52) are some of the most economically developed regions in China with high levels of car ownership. Traffic emissions were the main source of PAH emissions in summer in these regions. Traffic emissions of PAHs accounted for the highest proportion of total PAHs (25.2%) during summer in Bengbu city, although the high concentrations at this time of the year may also be related to PAHs brought into the area by the polluted monsoon.

The autumn PAH concentration in Bengbu was 5.91 ng/m^3^, and was heavily influenced by the pollutant concentrations resulting from cloud T2 (9.31 ng/m^3^) which originated from the north−west direction, including Shanxi, and cloud T3 (6.17 ng/m^3^) which originated from the north direction, including Hebei and Shandong. The pollutant concentrations from the southern Jiangsu airflow (Cloud T1) and the northern Jiangsu airflow (Cloud T4) had a lesser impact on Bengbu City, resulting in concentrations of 5.94 and 4.34 ng/m^3^, respectively. The areas with high PSCF scores in autumn were mainly located in the northern and northwestern regions, including Shanxi and Henan, which were also identified as potential pollution source areas for Bengbu City in autumn. Shanxi and Hebei provinces have relatively abundant coal resources, while there is also heavy use of coal in Shandong and Henan provinces (53). The proportions of atmospheric PAHs resulting from coal combustion in Chengde (41) and Zhengzhou (54) were 37.28 and 38.0%, respectively. Therefore, the pollution streams due to coal combustion in the northwestern parts of the city had a significant impact on the autumn PAH concentrations in Bengbu.

The PAH concentration in Bengbu in winter was 16.89 ng/m^3^. The T1 cloud from Shanxi, Hebei and Shandong and T3 cloud from Henan carried higher PAH concentrations (21.91 and 18.49 ng/m^3^) than the T2 cloud from Shandong and Jiangsu and the T4 cloud from Bohai and Jiangsu (13.51 and 14.05 ng/m^3^). The areas with high winter PSCF scores were also mainly located in the northern and north−western regions, such as Hebei, Henan, and Hubei. In the districts north of Huaihe River in China, large amounts of coal are burned for central heating in autumn and winter. The T1 and T3 air flows from the north−west, which carry coal combustion pollutants, compounded the PAH concentrations in Bengbu in winter. Wang J et al. (54) in Zhengzhou showed that the main source of the 16 priority PAHs was coal combustion, and Wang Y et al. (55) in Dalian found that the contribution of coal combustion to the PAH burden increased from 26 to 45% due to emissions from domestic heating in winter. Therefore, the long-range transport of pollutants from the northwest and north may have exacerbated winter PAH pollution in Bengbu.

The potential source areas derived from the PSCF analysis were the same as those derived from the backward trajectory clustering analysis, with Bengbu City being strongly influenced by the northwestern and northern regions in the spring, autumn, and winter, and strongly influenced by the monsoon from the eastern coastal region in the summer.

### 3.4 Health risk assessment

According to the USEPA, an ILCR lower than 1 × 10^−6^ can be regarded as negligible, and an ILCR above 1 × 10^−4^ is likely to be harmful to human beings. An ILCR value within a range of 1 × 10^−6^ to 1 × 10^−4^ indicates a tolerable risk for the public (46).

**Figure 6:**
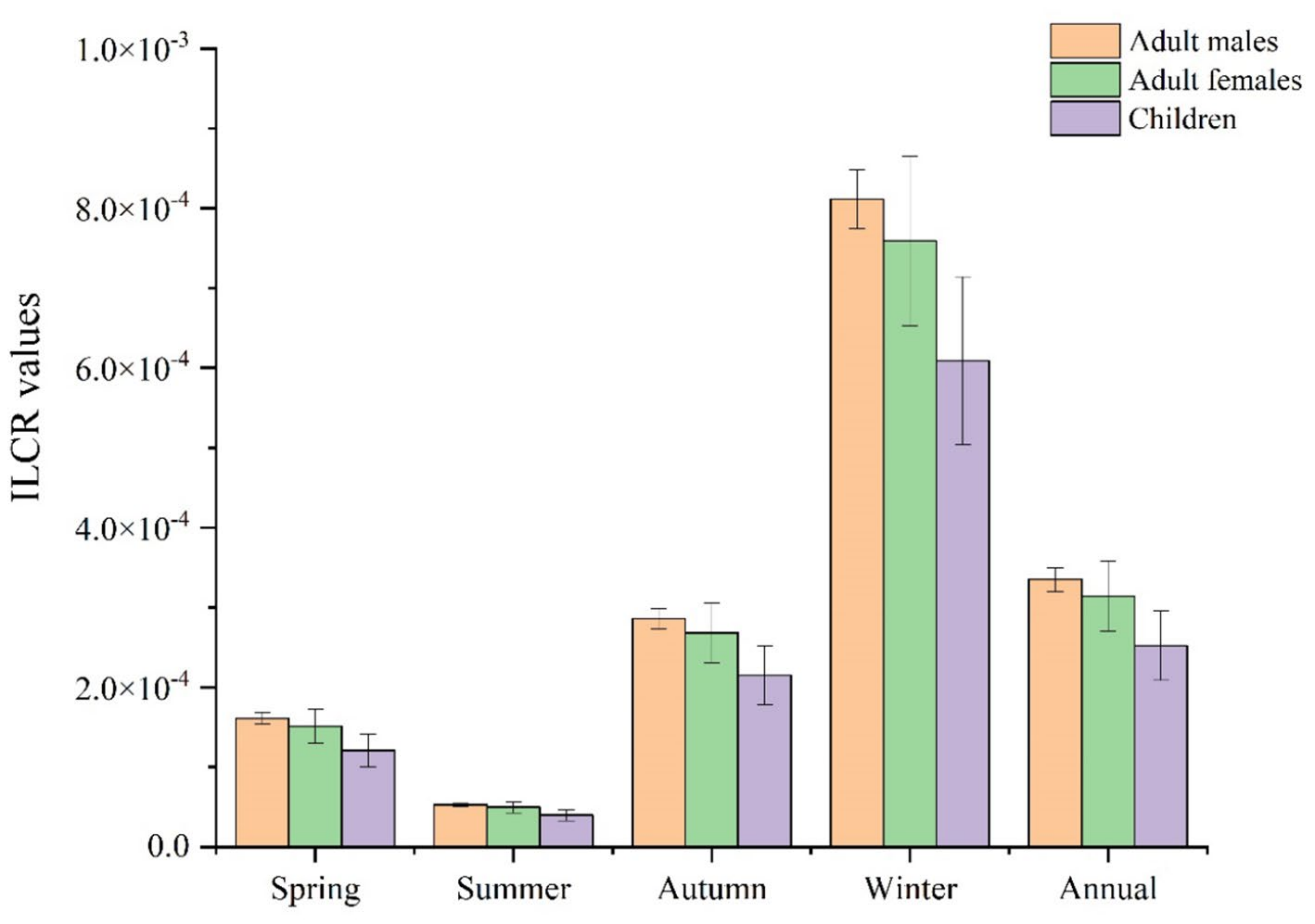
The ILCRs from PAH inhalation exposure in the four seasons.

As shown in Figure 6, the ILCR value of PAH exposure through inhalation for adult males was highest in winter (8.11 × 10^−4^), followed by autumn (2.86 × 10^−4^), spring (1.61 × 10^−4^), and summer (5.30 × 10^−5^), with an annual value of 3.35 × 10^−4^. The ILCR values for adult females and adult males in the four seasons were comparable, but slightly lower in adult females: winter (7.59 × 10^−4^) > autumn (2.68 × 10^−4^) > spring (1.51 × 10^−4^) > summer (4.97 × 10^−5^), with an annual value of 3.14 × 10^−4^. The ILCR values for children during the study period were winter (6.09 × 10^−4^) > autumn (2.15 × 10^−4^) > spring (1.21 × 10^−4^) > summer (3.98 × 10^−5^), with an annual value of 2.52 × 10^−4^. All categories of people were at risk in spring, autumn and winter, with adult males at higher risk than adult females, and all adults at higher risk than children, which was likely due to adults’ faster breathing rates and longer exposure times (56).

It was apparent that PAH pollution in Bengbu was more severe in spring, autumn, and winter, and the health risk to the population was therefore more severe at this time. The health risk for adult males was greater than that for adult females, and the health risk to adults was greater than that for children.

There is a certain amount of uncertainty in the risk assessment process at the population level. Changes in the environment in which the population is located at the time of the assessment may bring about changes in exposure pathways, and exposure concentrations may fluctuate over time. There are also limitations in the statistical models themselves, and there are also uncertainties in the parameters that are incorporated into the models during the assessment process.

## 4 Conclusion

Polycyclic aromatic hydrocarbons were measured in PM_10_ samples collected in four seasons at a site in Bengbu City. The annual concentrations of the 16 priority PAHs in Bengbu City ranged from 1.45 to 62.16 ng/m^3^, with an annual mean concentration of 7.63 ± 7.38 ng/m^3^. There was a seasonal trend for concentrations to be significantly higher in winter (6.13–62.16 ng/m^3^, median = 14.99 ng/m^3^) than in spring (1.45–19.34 ng/m^3^, median = 3.32 ng/m^3^), summer (1.57–4.27 ng/m^3^, median = 2.12 ng/m^3^), and autumn (2.01–18.78 ng/m^3^, median = 4.90 ng/m^3^). The annual average BaP concentration was 0.61 ng/m^3^, contributing 8.11% of ΣPAHs. The PAHs in the full year were dominated by MMW PAHs (39.81%), followed by HMW PAHs (35.77%) and LMW PAHs (24.42%).

The diagnostic ratio method and PMF revealed that the sources of PAH pollution in Bengbu in spring and summer were industrial emissions, coal and biomass combustion, and traffic emissions; while the sources of PAH pollution in autumn and winter were coal and biomass combustion and traffic emissions. According to the backward trajectory clustering analysis and PSCF potential source analysis, Bengbu City was strongly affected by pollution from the northern and northwestern regions in spring, autumn, and winter, while it was more affected by the coastal monsoon in summer.

The atmospheric PAH pollution in Bengbu was most severe in spring, autumn, and winter, and the health risk to the population was also most severe at that time. The health risk to adult males was greater than the risk to adult females, and the health risk to adults was greater than the risk to children.

## Data Availability

The original contributions presented in the study are included in the article/supplementary material, further inquiries can be directed to the corresponding author.

## Footnotes

^1^ http://www.noaa.gov

^2^ http://www.meteothinker.com
